# The Effect of Multipoint Injection Strategies of BMSCs on Repairing Cartilage Defects of the Knee Joint

**DOI:** 10.1111/jcmm.70978

**Published:** 2026-01-08

**Authors:** Wang Tang, Jiaqi Li, Yu Dai, Jiaxin Liang, Lei Wan

**Affiliations:** ^1^ Guangzhou Hospital of Integrated Traditional and Western Medicine Guangzhou China; ^2^ The Third Clinical Medical College of Guangzhou University of Chinese Medicine Guangzhou China; ^3^ Guangzhou University of Chinese Medicine Guangzhou China

**Keywords:** BMSCs, cartilage defects, cartilage injury, multipoint injection

## Abstract

Bone marrow‐derived mesenchymal stem cells (BMSCs) are extensively utilised in tissue engineering and regenerative medicine due to their multipotent differentiation capabilities. However, the therapeutic efficacy of BMSCs is highly dependent on the transplantation route. This study aimed to compare the efficacy of commonly used BMSCs transplantation methods and identify the optimal delivery approach for cartilage repair. Our results demonstrated that all transplantation methods could significantly suppress pro‐inflammatory factors, including IL‐1β, iNOS, and MMP‐9, while enhancing the activity of the key antioxidant enzyme superoxide dismutase (SOD). The intra‐articular injection group exhibited the most substantial anti‐inflammatory and antioxidant improvements. In vivo tracking experiments revealed that BMSCs from all groups were capable of homing to the cartilage defect site at 4 weeks post‐modelling. Notably, the intra‐articular injection group recruited the highest number of BMSCs to the defect area. Further histological analysis indicated that the joints treated with intra‐articular injection displayed superior cartilage regeneration, characterised by a smooth tissue surface and coloration closely resembling adjacent native cartilage. In conclusion, while all tested BMSCs transplantation approaches contributed to cartilage repair, intra‐articular injection demonstrated the most favourable therapeutic outcomes.

## 1 Introduction

Articular cartilage is a specialised form of hyaline cartilage, characterised by its absence of nerves, blood vessels, and lymphatic vessels. It functions to reduce friction, bear mechanical loads, and provide cushioning during joint movement. Owing to this avascular and aneural nature, articular cartilage exhibits a severely limited capacity for self‐repair following injury induced by biological factors or mechanical trauma [1, 2]. Cartilage injury triggers a progressive upregulation of various inflammatory genes associated with cartilage destruction. Key inflammatory mediators such as Interleukin‐1 beta (IL‐1β) and Inducible Nitric Oxide Synthase (iNOS) promote the expression of matrix metalloproteinases (MMPs) and aggrecanases, which collectively accelerate the degradation of extracellular matrix and the progression of osteoarthritis (OA) [3, 4].

With the advancement of cell‐based therapies, bone marrow‐derived mesenchymal stem cells (BMSCs) have garnered significant attention in regenerative medicine due to their abundant availability, ease of cultivation, and favourable histocompatibility [5]. BMSCs represent a promising therapeutic resource, capable of modulating the imbalance between anabolic and catabolic processes in OA through their immunomodulatory effects and regenerative potential [6]. The therapeutic actions of BMSCs are primarily mediated through paracrine signalling. They secrete various growth factors and immunomodulatory molecules, including members of the TNF‐β superfamily, which can counteract the catabolic effects of inflammatory cytokines such as tumour necrosis factor‐alpha (TNF‐α) and IL‐1β. Furthermore, extracellular vesicles released by BMSCs have also been demonstrated to play a crucial role in promoting cartilage regeneration [7, 8]. Multiple transplantation strategies for BMSCs have been reported, including local injection at the injury site, intravenous injection, intraperitoneal injection, and transplantation using tissue‐engineered scaffolds [9, 10, 11, 12]. While BMSCs delivered via different routes can generally home to survive within, and differentiate at the injury site, studies indicate significant variations in the extent of functional recovery achieved by these distinct approaches [11, 13, 14]. To identify the optimal delivery route for BMSCs in the context of cartilage defects, this study aimed to systematically evaluate and compare the therapeutic efficacy of intraperitoneal, ear vein, and intra‐articular injections of BMSCs on cartilage repair in a rabbit model.

## 2 Materials and Methods

### 2.1 Materials

Three‐month‐old female New Zealand white rabbits, with a mean body weight of 2.0 ± 0.2 kg, were supplied by the Animal Experimental Centre of Guangzhou University of Chinese Medicine. All animals were housed under standard laboratory conditions with ad libitum access to food and water. The rabbits were randomly assigned into five experimental groups (*n* = 5 per group): (1) a blank control group, (2) a model control group, (3) an intraperitoneal injection group, (4) an ear vein injection group, and (5) an intra‐articular injection group. The rabbit BMSC culture medium, chondrogenic induction medium, and osteogenic induction medium were commercially procured from Cyagen Biosciences (Guangzhou, China). The study protocol was reviewed and approved by the Ethics Committee of Guangzhou Integrated Traditional Chinese and Western Medicine Hospital, ensuring compliance with all relevant guidelines for the ethical use of animals in research.

### 2.2 Cell Culture and Identification

BMSCs were isolated using the whole bone marrow adherence method. The bone marrow was cultured in complete rabbit bone marrow mesenchymal stem cell medium supplemented with 10% fetal bovine serum and 1% penicillin–streptomycin in a humidified incubator at 37°C with 5% CO_2_. The first medium change was performed with half‐volume replacement after 72 h, and subsequent changes were carried out every 2–3 days thereafter depending on cell growth. When cells reached approximately 90% confluence, they were detached using 0.25% trypsin and subcultured at a 1:3 ratio. Third passage (P3) cells were seeded into 12‐well plates at a density of 1 × 10^4^ cells per well. After cell attachment, the culture medium was replaced with either chondrogenic or osteogenic induction medium. The induction medium was refreshed every 2 days for 14 days, after which the chondrogenic and osteogenic differentiation potentials were assessed by Alcian blue staining, Alizarin red S staining, and Alkaline Phosphatase (ALP) staining, respectively. Images were captured using an inverted microscope equipped with a camera.

### 2.3 Cartilage Defect Modelling and Grouping

Twenty three‐month‐old New Zealand white rabbits were anaesthetised with 3% pentobarbital sodium (30 mg/kg) by slow intravenous injection along the ear edge. The skin of the rabbits’ knees was prepared and disinfected with iodine tincture. Disinfectant towels were applied in the operative area after alcohol de‐iodisation. A lateral parapatellar incision was made for the right knee, and the skin, subcutaneous tissue, and joint capsule were incised to expose the lateral condyle of the right femur. A hole of 4 mm in diameter and about 3 mm in depth was drilled in the anterior middle third of the lateral condyle, and fresh blood was seeping into the joint cavities, resulting in a full‐thickness articular cartilage injury that involved the subchondral area. The incision was closed layer by layer after normal saline irrigation. Five rabbits in the blank group only opened the joint cavity and were rinsed with normal saline. After the operation, rabbits were divided into cages, and each rabbit was given an intramuscular injection of penicillin 400,000 units twice before and 3 days after the operation.

### 2.4 Cartilage Defect Model and BMSCs Injection

One week after the operation, the 20 model animals were randomly divided into the model group, intraperitoneal injection group, ear vein injection group, and intra‐articular injection group. The other five animals that were not subjected to modelling served as the blank group. A single dose of 2 × 10^5^ BMSCs in 0.2 mL saline was administered according to the respective route for each treatment group. The model group received no intervention, while the blank group served as the untreated baseline. Injections were administered once per week for two consecutive weeks. All BMSCs were labelled with BrdU prior to injection. The rabbits were housed in separate hutches and allowed to move freely.

### 2.5 Measurement of Inflammatory Mediators in Synovial Fluid

At the 4 week post‐intervention time point, approximately 0.5 mL of synovial fluid was aspirated from the knee joint of each rabbit. The samples were immediately centrifuged at 1500 × g for 10 min to remove cells and debris. The resulting supernatants were aliquoted and stored at −70°C until subsequent analysis. The concentrations of specific inflammatory mediators and an antioxidant enzyme—namely interleukin‐1β (IL‐1β), matrix metalloproteinase‐9 (MMP‐9), iNOS, and SOD—were quantified using commercially available enzyme‐linked immunosorbent assay (ELISA) kits, strictly in accordance with the manufacturers’ protocols.

### 2.6 ICRS Histological Scoring and Brdu Immunohistochemistry Detection

BMSCs at passage three were seeded in 12‐well culture plates. When the cells grew to 60 BMSCs at passage three were seeded in 12‐well culture plates. When the cells grew to 60% confluence, the medium was aspirated and replaced with fresh medium containing 10 μM 5‐bromo‐2‐deoxyuridine (BrdU) for 48 h. Subsequently, the cells were fixed with cold 70% ethanol for 5 min at room temperature. After blocking with 5% normal goat serum, the cells were incubated with a primary BrdU antibody overnight at 4°C. Following PBS washes, the cells were incubated with a fluorochrome‐conjugated secondary antibody for 1 h at room temperature in the dark. Nuclei were counterstained with DAPI‐containing Prolong Gold AntiFade Reagent. Fluorescence microscopy was used to determine the BrdU labeling efficiency. BrdU‐positive cells were quantified by counting DAPI‐positive nuclei and BrdU‐positive nuclei in five randomly selected high‐power fields (400 × magnification) per sample using ImageJ software. The labeling efficiency was calculated as the percentage of BrdU‐positive nuclei relative to the total DAPI‐positive nuclei.

At 4 weeks post‐surgery, the distal femurs from all groups were harvested. The quality of cartilage repair was assessed by two double‐blinded researchers using the ICRS histological scoring system. The regenerated tissues were fixed in 4% paraformaldehyde for 48 h, decalcified in 10% EDTA for 4 weeks, and then embedded in paraffin and sectioned. After deparaffinisation and rehydration, antigen retrieval was performed using citrate buffer in a microwave. Endogenous peroxidase activity was quenched with 3% hydrogen peroxide. The sections were then incubated with BrdU primary antibody overnight at 4°C, followed by incubation with a secondary antibody. Finally, the sections were washed, coverslipped, and examined under an inverted phase contrast microscope. Positively stained BMSCs were quantified in three random areas of each section. Additionally, sections were subjected to H&E staining and Toluidine Blue staining.

### 2.7 Statistical Analysis

All quantitative data are presented as mean ± standard deviation (SD). Normality of data distribution was confirmed using the Shapiro–Wilk test. Statistical analysis was performed using the SPSS 24.0 software (version 9.0.0, GraphPad Software, USA). For comparisons among multiple groups, a one‐way analysis of variance (ANOVA) was conducted, followed by Tukey's honest significant difference (HSD) post hoc test for pairwise comparisons. Data normalisation was applied where necessary to ensure comparability. The threshold for statistical significance was set at *p* < 0.05. In addition to *p*‐values, the effect size was calculated to interpret the practical significance of the findings. For the overall ANOVA model, partial eta‐squared (*η* ^*2*^) was reported and interpreted as follows: small (*η* ^*2*^ ≥ 0.01), medium (*η* ^*2*^ ≥ 0.06), and large (*η* ^*2*^ ≥ 0.14). For significant post hoc pairwise comparisons, Cohen's *d* was calculated and interpreted as: small (*d* ≥ 0.2), medium (*d* ≥ 0.5), and large (*d* ≥ 0.8).

## 3 Results

### 3.1 Identification of BMSCs

**FIGURE 1:**
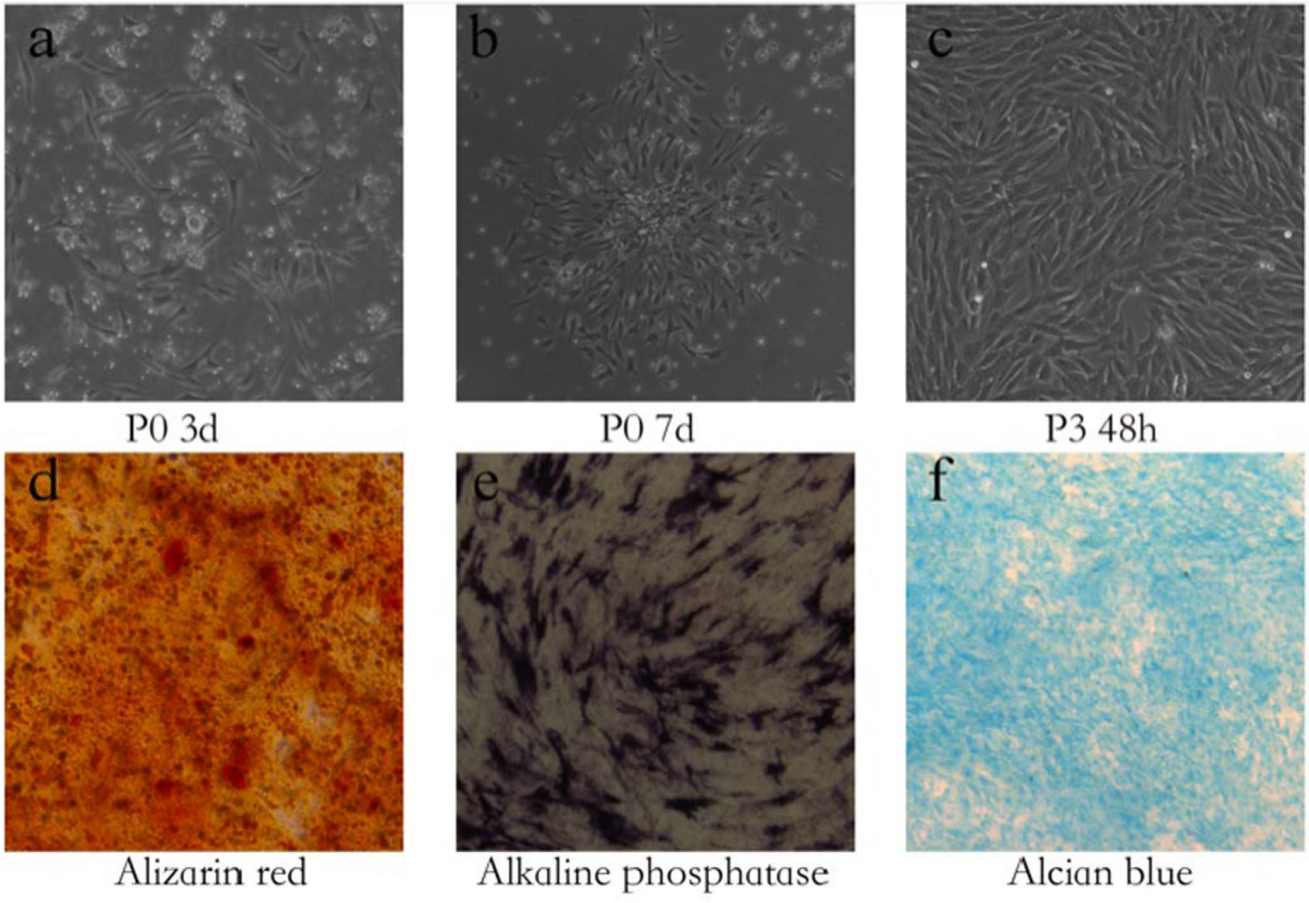
Characterisation of rabbit bone marrow‐derived mesenchymal stem cells. (a) Primary culture showing adherent, fibroblast‐like cells with spindle‐shaped morphology. (b) Cell colonies with varying sizes and morphologies formed during expansion. (c) Cells reaching approximately 90% confluence after 72 h of culture. (d) Osteogenic differentiation demonstrated by Alizarin Red S staining, showing calcium deposition (red nodules) after 2 weeks of induction. (e) Osteogenic differentiation confirmed by ALP staining (purple reaction product) after 2 weeks of induction. (f) Chondrogenic differentiation revealed by Alcian blue staining, indicating proteoglycan synthesis (blue matrix) after 2 weeks of induction.

Bone marrow was aseptically harvested from healthy New Zealand white rabbits. Primary cells were cultured in vitro, and adherent, spindle‐shaped cells exhibiting active proliferation capacity were observed after 72 h of culture. To characterise the multipotent differentiation potential of the isolated cells, osteogenic and chondrogenic induction assays were performed. Following osteogenic induction, the majority of cells demonstrated osteogenic differentiation capability, as evidenced by positive staining with Alizarin Red S for mineralization and ALP for early osteogenic activity. In parallel, chondrogenic induction led to positive Alcian blue staining, confirming the synthesis of proteoglycans characteristic of chondrogenic differentiation. These results collectively verified that the isolated adherent cells possessed the defining characteristics of BMSCs, including plastic adherence and multipotent differentiation capacity, after three passages (Figure 1).

### 3.2 Effect of BMSCs on Inflammatory and Antioxidant Profiles in Synovial Fluid

**FIGURE 2:**
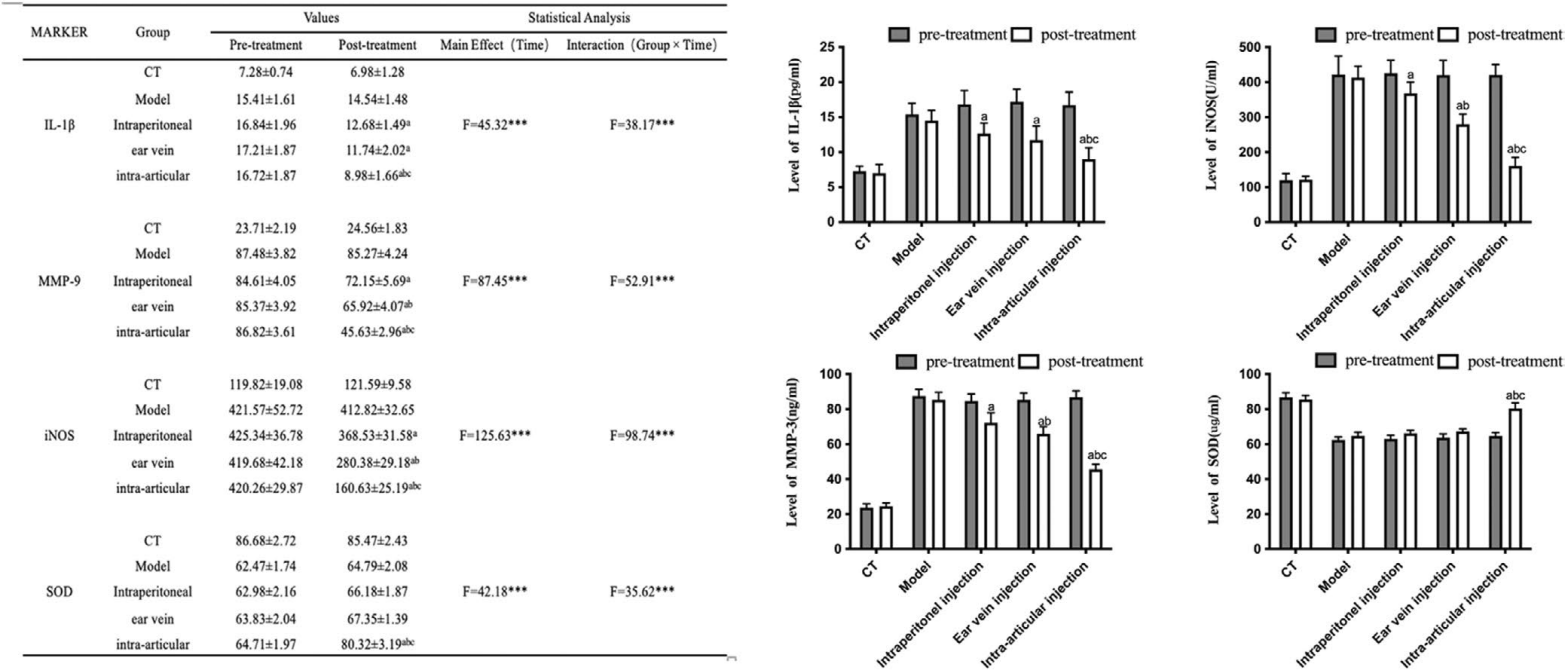
Comparisons of inflammatory factors in the synovial fluid of rabbit right knee joints between groups. Concentrations of (A) IL‐1β, (B) MMP‐9, (C) iNOS, and (D) SOD were measured in synovial fluid before and after treatment across different groups. Data are presented as mean ± SD. Statistical significance was determined by two‐way ANOVA followed by Tukey's post‐hoc test. Effect sizes (partial *η* ^*2*^) for the main effect of Group were as follows: IL‐1β = 0.83, MMP‐9 = 0.89, iNOS = 0.92, SOD = 0.79. Superscript letters indicate significant differences in post hoc comparisons: *p < 0.05 versus Model group; **p < 0.05 versus Intraperitoneal group; ***p < 0.05 versus Ear‐vein group. ***p < 0.001 for the main effect of Time and the Group × Time interaction.

Compared to the blank control group, the model group exhibited significantly elevated levels of pro‐inflammatory mediators, including IL‐1β, MMP‐9, and iNOS (*p* < 0.05), alongside a significant reduction in the antioxidant enzyme SOD (*p* < 0.05), confirming the successful induction of an inflammatory microenvironment following the cartilage defect. Administration of BMSCs via all tested routes significantly attenuated this inflammatory response. All treatment groups showed a marked reduction in the concentrations of IL‐1β, MMP‐9, and iNOS compared to the model group (*p* < 0.05). Notably, the intra‐articular injection group showed the most pronounced suppression of these pro‐inflammatory factors. Conversely, SOD activity was significantly restored following BMSCs transplantation (*p* < 0.05). This enhancement in antioxidant capacity was most evident in the intra‐articular injection group (*p* < 0.05), consistent with its superior anti‐inflammatory effects (Figure 2).

### 3.3 Homing of BrdU‐Labelled BMSCs to Cartilage Defect Sites

**FIGURE 3:**
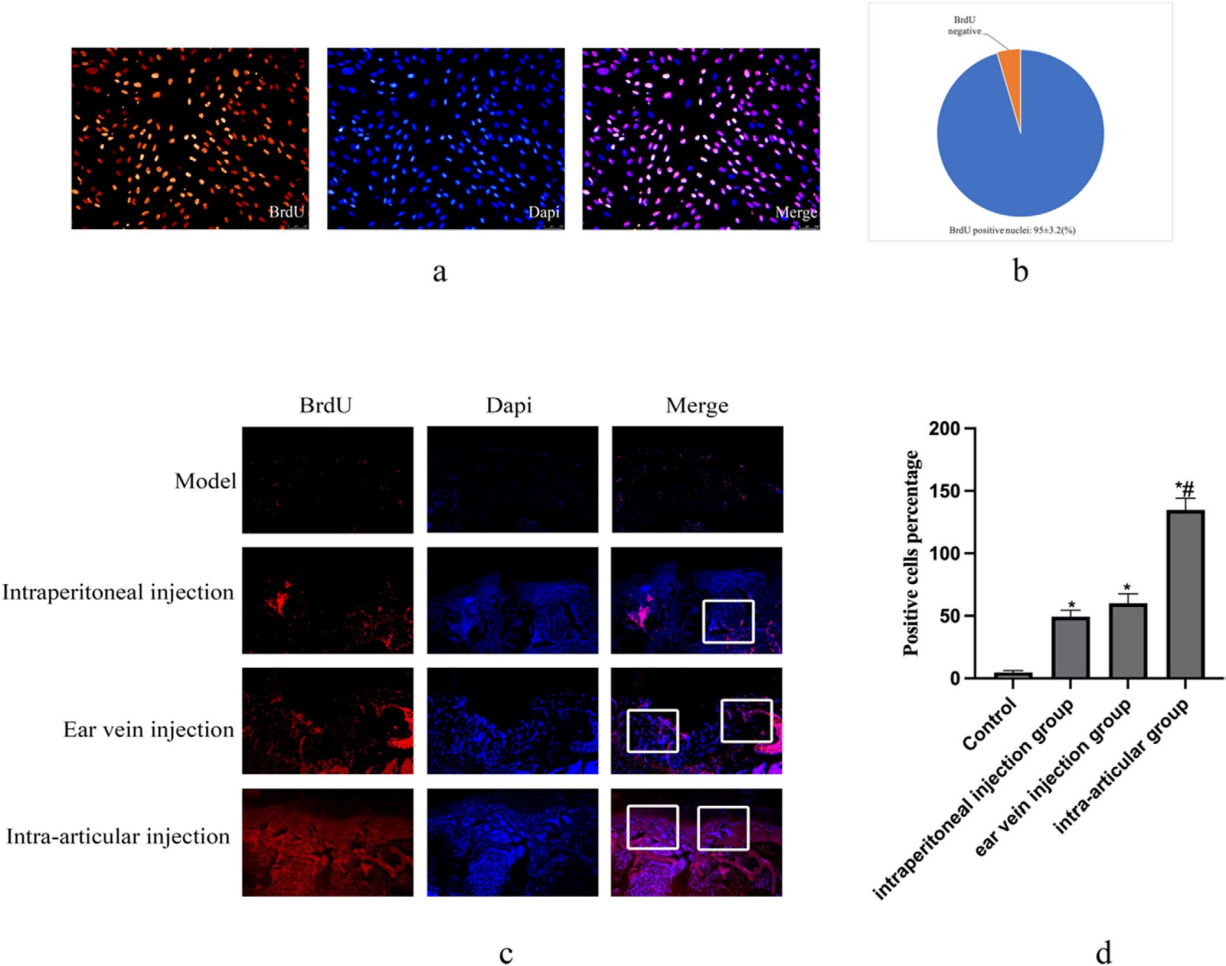
The migration of Brdu‐labelled BMSCs in vivo. (a) To monitor the in vivo migratory behaviour of BMSCs, BMSCs were marked with BrdU. All cell nuclei stained with DAPI manifested as blue, while BrdU‐positive cells presented with red fluorescence. (b) The labelling efficiency of BrdU was 95% ± 3.2%. (c) BrdU‐positive cells were evaluated via an immunofluorescence assay at 4 weeks following surgery. The count of BMSCs in intra‐articular group within the repairing tissue was conspicuously higher than that in the group inoculated with control BMSCs. (d) Statistical data analysis of the positive cell percentage in Figure 3c. Data are presented as mean ± SD. Statistical analysis was performed using two‐way ANOVA followed by Tukey's post hoc test. *p < 0.05 compared with the Model. **p < 0.05 compared with the other groups.

To track the transplanted cells, BMSCs were labelled with BrdU prior to administration. Immunofluorescence assay confirmed a high labelling efficiency of 95.2% ± 3.2% after incubation with 10 μm BrdU for 72 h (Figure 3a,b). The homing of BrdU‐labelled BMSCs to the cartilage defect sites was assessed 4 weeks post‐surgery. As shown in Figure 3c,d, the number of BrdU‐positive cells within the repair tissue varied significantly among groups. The intra‐articular injection group exhibited a significantly higher number of BrdU‐positive cells at the defect site compared to all other delivery routes, with a more extensive and uniform distribution throughout the repair tissue. These results indicate that intra‐articular injection provides the most efficient delivery and retention of BMSCs at the site of cartilage injury.

### 3.4 Histological Evaluation of Cartilage Repair Efficacy

**FIGURE 4:**
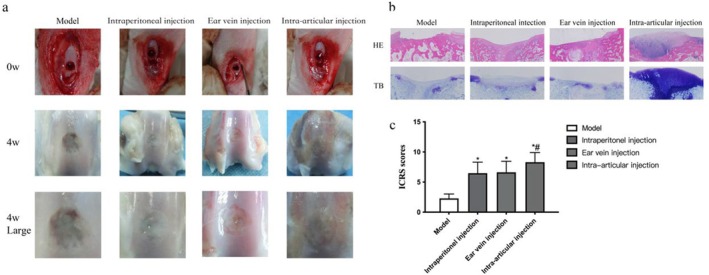
Macroscopic and histological evaluation of cartilage repair at 4 weeks post‐surgery. (a) Representative macroscopic views of cartilage defects. The intra‐articular injection group displays superior repair quality with smooth surface integration (arrows) and native‐like coloration. (b) Corresponding H&E and Toluidine Blue staining. The intra‐articular injection group shows well‐structured cartilage restoration with abundant proteoglycan deposition (intense metachromatic staining). (c) Quantitative ICRS macroscopic scores. Data represent mean ± SD (*n* = 5). *p < 0.05 versus Model group; **p < 0.05 versus both Intraperitoneal and Auricular intravenous groups (one‐way ANOVA with Tukey's test).

Histological evaluation further elucidated the differential repair outcomes among groups. H&E staining revealed substantial cartilage disruption in the model group, characterised by surface fissures and reduced thickness (Figure 4b). A clear treatment gradient was observed: the intraperitoneal injection group showed partial improvement, the ear vein injection group demonstrated more substantial repair, and the intra‐articular injection group exhibited near‐normal cartilage architecture, with optimal surface regularity and thickness restoration closely resembling native tissue (Figure 4a, c). Toluidine blue staining confirmed these findings, showing significantly enhanced proteoglycan content in all treatment groups compared to the model group, with the most intense and homogeneous ECM staining observed in the intra‐articular injection group (Figure 4b).

## 4 Discussion

This study demonstrates that while multiple BMSCs delivery routes contribute to cartilage repair, intra‐articular injection yields the most favourable therapeutic outcomes, as evidenced by superior histological scores, enhanced anti‐inflammatory effects, and improved cell homing. However, this efficacy is tempered by the unique risk of chondroma‐like formation, highlighting a critical safety consideration for clinical translation.

The repair of articular cartilage defects remains a significant clinical challenge due to the tissue's limited self‐repair capacity [9, 15, 16]. Our findings align with the established dual role of BMSCs in regenerative medicine, encompassing both direct structural contribution and potent paracrine immunomodulatory effects [17, 18]. The significant reduction in pro‐inflammatory mediators (IL‐1β, iNOS, MMP‐9) and upregulation of SOD across all treatment groups substantiate this systemic anti‐inflammatory capacity. Notably, the persistent inflammatory state in the blank control group establishes a baseline of failed natural healing, against which therapeutic efficacy can be rigorously evaluated.

The superior efficacy of intra‐articular injection can be attributed to its unique ability to establish a concentrated ‘therapeutic niche’ at the injury site. This route achieves optimal pharmacokinetic advantage through first‐pass localization, ensuring maximum cellular bioavailability. Our BrdU‐tracking data directly confirm that intra‐articular delivery results in significantly higher cell retention and more homogeneous distribution within the defect, a critical factor for effective regeneration. This strategic localization enables BMSCs to secrete a complex cocktail of trophic factors (e.g., TGF‐β, IGF‐1, HGF) at high concentrations, profoundly modulating the local microenvironment [19]. This concentrated paracrine action is reflected in our findings of the most substantial suppression of pro‐inflammatory mediators and restoration of SOD activity in the intra‐articular group. Beyond inflammation control, this niche facilitates essential regenerative processes: Enhanced subchondral angiogenesis, suppression of hypertrophic differentiation, and promotion of progenitor cell migration—all vital for functional cartilage restoration [20]. The resulting microenvironment, characterised by balanced anabolic‐catabolic signalling and reduced oxidative stress, optimally supports chondrogenic differentiation and matrix synthesis, ultimately manifesting as the superior histological outcomes observed.

In contrast, systemic delivery routes face substantial biological barriers. Intravenous injection subjects cells to pulmonary first‐pass sequestration, drastically reducing systemic circulation [21]. while the avascular nature of cartilage further impedes cell infiltration. Intraperitoneal injection, despite accommodating large cell boluses, results in unpredictable dispersal and omental entrapment, severely compromising targeted homing [22]. The minimal repair in control groups accentuates that even modest improvements from systemic delivery represent significant advances over natural disease progression, though these outcomes remain inferior to localised approaches.

**FIGURE 5:**
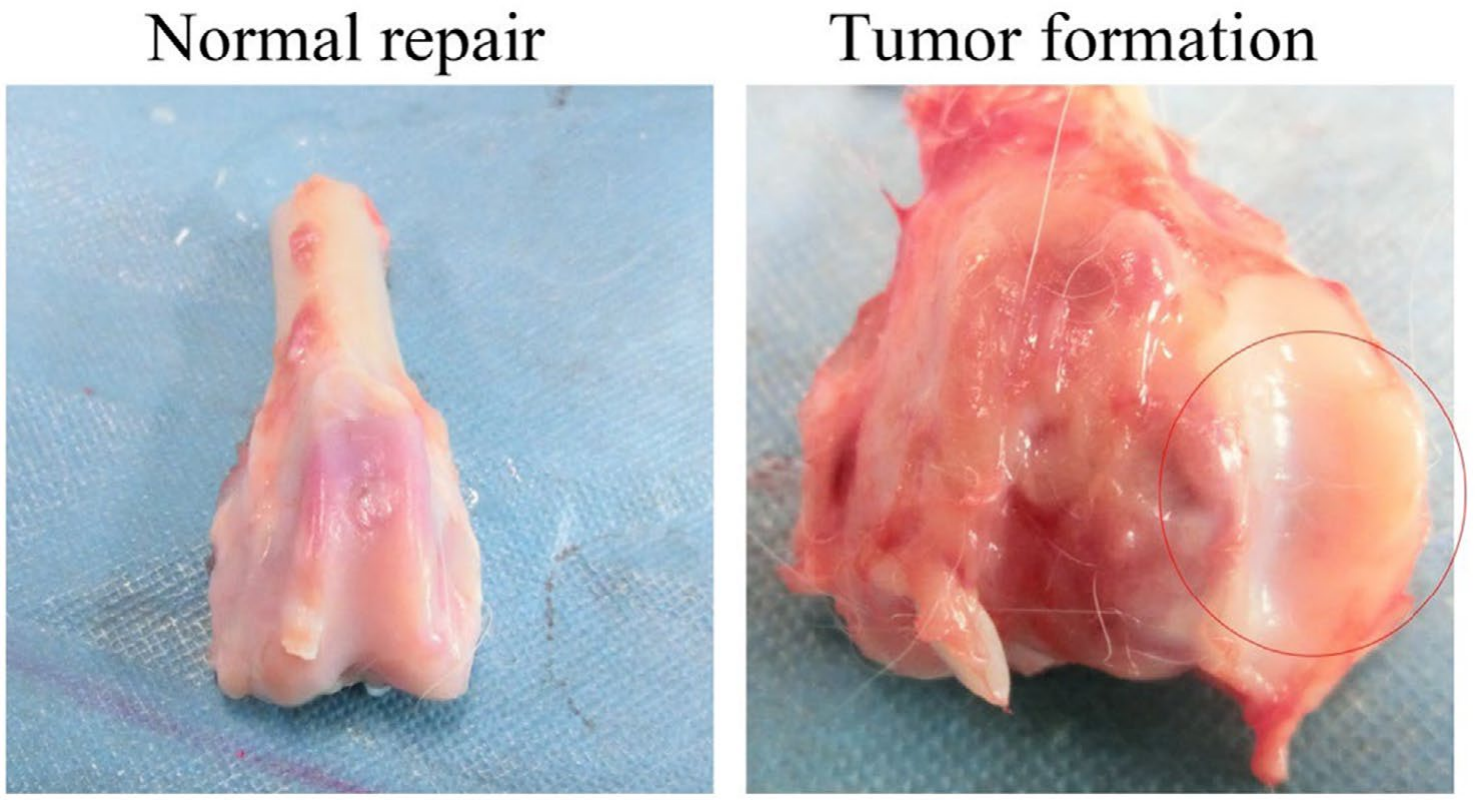
Abnormal tissue formation after intra‐articular injection of BMSCs: Representative images demonstrating successful cartilage repair following BMSCs transplantation, characterised by smooth surface continuity and seamless integration with the surrounding native tissue. Representative images revealing a safety concern associated with intra‐articular BMSCs injection. A macroscopic view shows a distinct, nodular outgrowth (circle) on the articular surface. This aberrant tissue formation was observed exclusively in a subset of animals within the intra‐articular injection group.

The most clinically significant finding of our study is the paradoxical association between maximal efficacy and unique risk—the exclusive formation of chondroma‐like structures in the intra‐articular group. The absence of such proliferation in blank controls definitively links this phenomenon to BMSC therapy rather than spontaneous tumorigenesis (Figure 5). We hypothesize that the unconstrained synovial environment, unlike the structured defect niche, permits formation of high‐density cell aggregates where loss of contact inhibition and dysregulated autocrine/paracrine signalling—potentially involving TGF‐β and BMP pathways—drive uncontrolled proliferation over organised chondrogenesis [23, 24]. Additionally, high‐concentration cell injection may induce transient synovitis [25], creating an inflammatory milieu that promotes hyper‐proliferation rather than phased differentiation [26]. This underscores that therapeutic outcomes are governed not only by cellular intrinsic properties but equally by the implantation niche.

Our findings have immediate implications for the clinical translation of BMSC therapies. They strongly caution against the simple intra‐articular injection of high doses of naive BMSCs. Future work must focus on strategies to harness the efficacy of intra‐articular delivery while mitigating the risk of abnormal tissue growth. Several promising approaches emerge: Biomaterial‐Guided Delivery: The use of biocompatible hydrogels or scaffolds can physically confine cells to the defect site, providing a three‐dimensional matrix that guides organised tissue formation and prevents cell dispersion and aggregation in the joint space [27, 28]. Pre‐differentiation of MSCs: In vitro pre‐differentiation of BMSCs into chondroprogenitors prior to implantation could generate a more committed and stable cell population, less prone to aberrant proliferation in response to joint environmental cues [29]. Dosage and Formulation Optimization: A critical, dose‐dependent threshold for safe cell delivery must be established. Furthermore, the use of smaller, more frequent injections or cells suspended in viscous carriers could prevent the formation of large, problematic cell clumps. Modulation of the Joint Microenvironment: Co‐delivery of BMSCs with anti‐inflammatory agents (e.g., IL‐1 receptor antagonist) or specific small‐molecule inhibitors that fine‐tune the Wnt or TGF‐β signalling pathways could help steer differentiation toward stable hyaline cartilage and away from hypertrophic or neoplastic phenotypes [30].

This study has certain limitations that should be acknowledged. Firstly, while our experimental design included essential blank and model control groups to benchmark the final therapeutic outcome, we did not measure the inflammatory cytokine levels immediately after defect creation and prior to BMSCs treatment. The inclusion of such pre‐intervention baseline data would have provided a more complete picture of the dynamic inflammatory changes and more precisely quantified the anti‐inflammatory effect of each treatment. Nevertheless, the post‐treatment cytokine data presented here robustly capture the differential joint microenvironments that are directly associated with the long‐term repair outcomes. Secondly, the safety concern regarding abnormal tissue growth, while critical, was observed in a specific model setting, and its clinical relevance requires further investigation across different species and defect conditions.

## 5 Conclusion

In conclusion, the poor regenerative outcome in the blank control group firmly establishes the therapeutic necessity for intervention. While our study unequivocally identifies intra‐articular injection as the most effective route for BMSC delivery in cartilage repair, it simultaneously reveals a critical safety concern. The progression of BMSC therapy from the bench to the bedside will depend on developing next‐generation delivery strategies that provide spatiotemporal control over cell behaviour, ensuring both robust regeneration and long‐term safety.

## Author Contributions

**Wang Tang:** writing – review and editing.

## Funding

This study was supported by Guangzhou Huadu District Science and Technology Program Project (23‐HDWS‐050).

## Conflicts of Interest

The authors declare no conflicts of interest.

## Data Availability

The data that support the findings of this study are available from the corresponding author upon reasonable request.
